# Toward the World Code Against Cancer

**DOI:** 10.1200/JGO.17.00145

**Published:** 2018-04-02

**Authors:** Carolina Espina, Rolando Herrero, Rengaswamy Sankaranarayanan, Etienne Krug, Christopher P. Wild, Joachim Schüz

**Affiliations:** **Carolina Espina**, **Rolando Herrero**, **Rengaswamy Sankaranarayanan**, **Christopher P. Wild**, and **Joachim Schüz**, International Agency for Research on Cancer, Lyon, France; and **Etienne Krug**, World Health Organization, Geneva, Switzerland.

## Abstract

Overwhelmed by an abundance of often confusing, ambiguous, or apparently contradictory messages on disease prevention in today’s multiple media streams, the general public would surely value authoritative, clear, and evidence-based instructions on how to actively contribute to the reduction of their cancer risk. The European Code Against Cancer is a set of 12 recommendations for individuals on how to reduce cancer risk. The Code carries the authority and reliability of expert scientists working under the coordination of the International Agency for Research on Cancer, the cancer research agency of the WHO. The Code’s messages are aimed at individuals and have been enthusiastically promoted by European cancer associations. The experience of developing and promoting the European Code has generated interest in developing analogous recommendations for other regions of the world. Under the overall umbrella of a World Code Against Cancer using the same International Agency for Research on Cancer methodology, regional Codes could be developed, focused on regions sufficiently large and distinct to merit development of versions adapted to regional differences in risk factors and cancer patterns. Consideration of such an adapted model illustrates why a simple translation of the European Code would not be sufficient to promote cancer prevention globally.

## BACKGROUND

**Fig 1:**
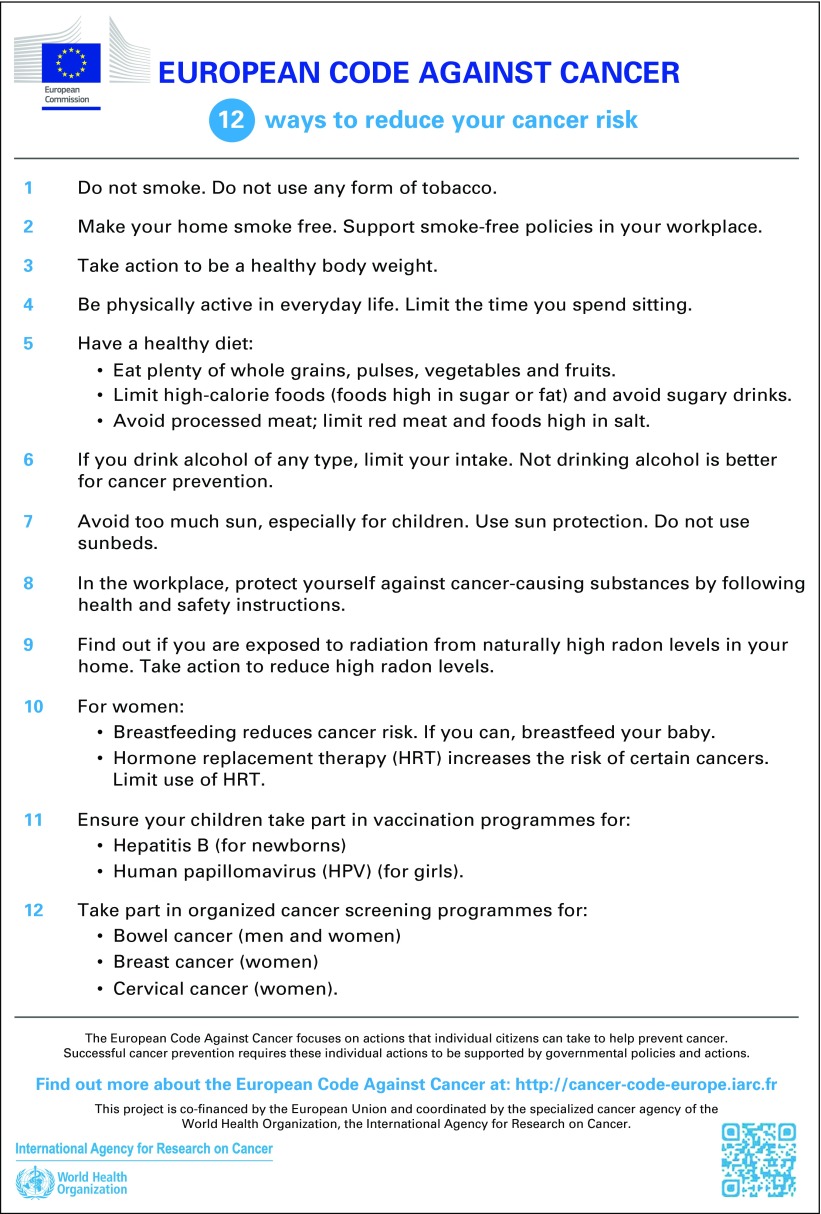
European Code Against Cancer, 4th edition.

It has been estimated that the cancer burden could be reduced by up to one half through primary and secondary prevention.^1^ The European Code Against Cancer (ECAC) is a unique multirisk prevention tool translating scientific knowledge on causes of cancer into actions individuals can take for cancer prevention. The ECAC was created in 1987 as a set of recommendations providing such advice on cancer prevention to European Union (EU) citizens. It informs the general public how to avoid or reduce exposures to scientifically established human carcinogens, adopt behaviors to reduce cancer risk, and participate in organized intervention programs under the appropriate national guidelines. It also informs stakeholders to guide national health policies in cancer prevention. The fourth edition was published in 2014 (Fig 1)^2^ and is accompanied by a compilation of questions and answers for the general public that further explain the recommendations, give instructions on how to best follow them, and clarify additional information on other exposures and topics of interest in cancer prevention, such as messages for special target groups.^3^

This tool, focused on the European situation, nevertheless provides an excellent basis for scaling up to a World Code Against Cancer, composed of adapted versions of the Code for different, defined regions of the world. Provision of science-based recommendations as a key cancer prevention tool should be tailored to the specific risk factors, cancer burden, and prevention priorities for the region. One of the features of the European Code was its development by leading cancer experts from the EU, helping ensure the focus was on the most relevant factors and encouraging ownership and application in the target population. For these reasons, simple translation of the European Code into other languages for use in other regions of the world would be insufficient. On the contrary, we consider it of utmost importance to adapt the Code to regional settings, cancer patterns, and health systems. Likewise, the most appropriate dissemination strategies should be designed to respond to regional circumstances, focusing on the most effective selected target groups (eg, the general public, health professionals, educators). Despite these important nuances, the experience from Europe provides the methodology and tools that are ready to use for the development of guidelines in other regions of the world, promising added value from the investment already made and the experience gained.

Successful preventive interventions require a combination of individual action (by avoiding or reducing harmful exposures or by making the decision to partake in a medical intervention) and public health policies and actions by national governments (when exposure is eliminated or reduced by effective and equitably accessible preventive measures at the population level). For individuals to engage in successful reduction of their cancer risk, they need to be informed about evidence-based actions in clear, unambiguous, and—although scientifically accurate—easy-to-understand language and how to put them into practice. Many of these actions, even at the individual level, need to be enabled by political, sociocultural, and economic considerations. The International Agency for Research on Cancer (IARC), the cancer research agency of WHO, has developed a rigorous evidence-based methodology to synthesize the scientific evidence, leading to the update of the ECAC,^4^ now as the fourth edition (Fig 1). Taking into account the most up-to-date and best available scientific data on causation and prevention of cancer, several working groups of cancer experts and, importantly, experts in communication of health messages, were established accordingly in 2012 to revise the existing recommendations. A Scientific Committee of leading experts on cancer prevention in the EU was established to review and approve the recommendations, ensuring broad support from the most authoritative sources and fostering broadest support of EU member countries. For extensions of the Code to other regions, it is equally essential to obtain the engagement, inputs, and support from the regional leaders in cancer prevention. In the European context, the criteria for selecting each recommendation and communicating the recommendations to the general public are shown in Table 1.^4^

**Table 1:**
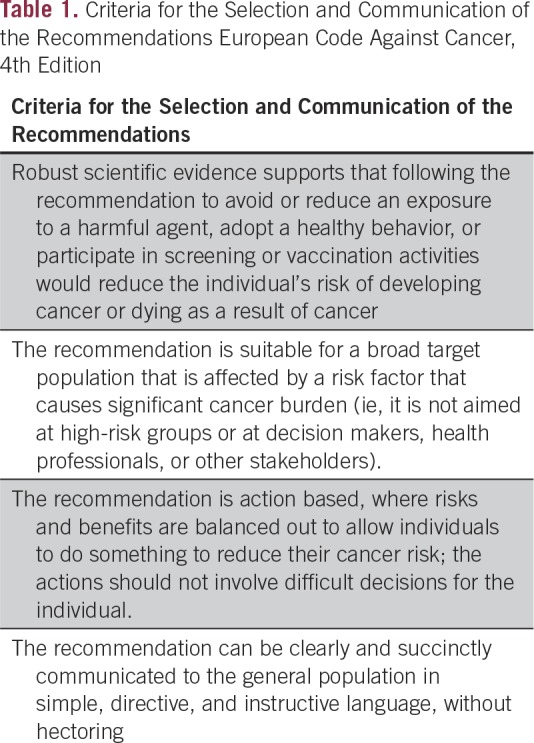
Criteria for the Selection and Communication of the Recommendations European Code Against Cancer, 4th Edition

## RATIONALE

**Fig 2:**
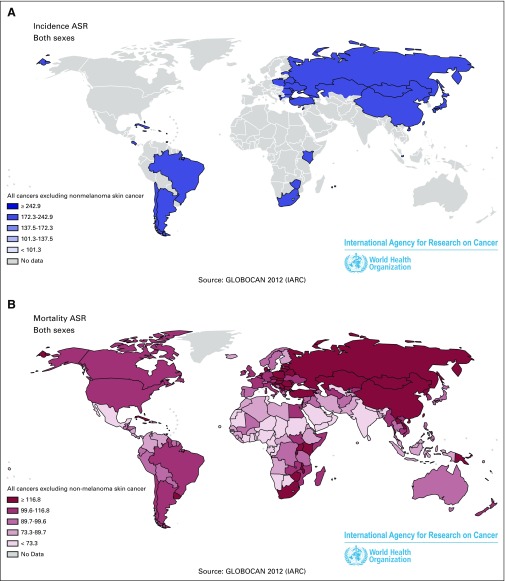
(A) World cancer incidence. (B) World cancer mortality. ASR, Age-standardized rate.

Cancer is the second-leading cause of death worldwide (14 million new cancer cases and 8.2 million cancer deaths in 2012; Figs 2A and 2B),^5^ and rapid population growth, changing lifestyles, and aging will result in a dramatic increase of the cancer burden by 2030. At the same time, low- and middle-income countries will continue to encounter serious challenges, such as wide inequities, limited financial resources, poor infrastructure and education, densely populated cities or remote inaccessible regions, and segments of society that are socioeconomically disadvantaged (eg, indigenous populations). On the other hand, these same countries continue to grow at a fast pace, often shifting lifestyles toward an increase in smoking, unhealthy dietary habits, alcohol consumption, and sedentary behaviors. As a result, many of those countries experience a double burden of cancer, with infection-related cancers typically associated with poverty still being common,^6^ combined with an increasing incidence of lifestyle-associated cancers, such as lung, breast, and colorectal cancer, previously more common in high-income countries. This places a major strain on the already stretched and limited health care systems, often characterized by inadequate infrastructure, human resources, and poor access to cancer care. The main goal of developing regional versions of the ECAC would be to raise awareness about risk factors and available prevention measures by effectively communicating the current state of the science and, as a consequence, empowering the individual and the community. Differences compared with the European context in existing sociocultural norms, risk factor patterns, cancer burden, and the state of development of health systems necessitate an adaptation of the core set of recommendations for application in other areas of the world. In the following sections, we give several examples as to why adaptation is essential (for the purpose of this publication, we will not focus in North America or Oceania, where we envisage more similar recommendations as for the EU).

## REGIONAL SCENARIOS

### Cancer Patterns

#### Latin America.

**Fig 3:**
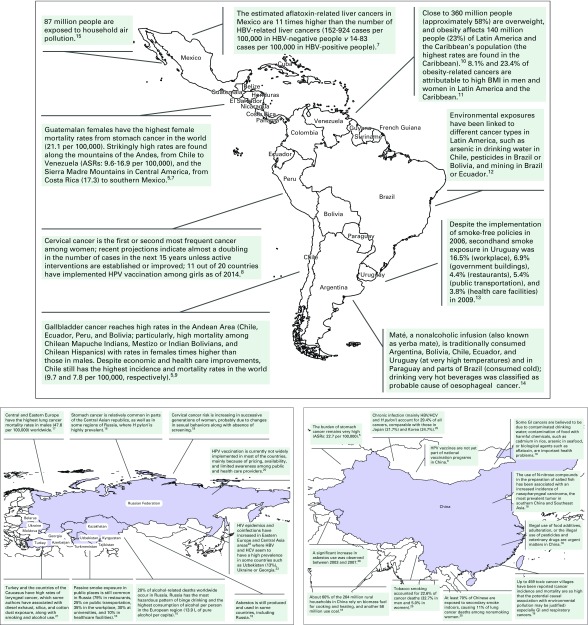
(A) Examples of risk factors and cancer patterns in Latin America. (B) Examples of risk factors and cancer patterns in non-EU Eastern European countries and former Soviet republics. (C) Examples of risk factors and cancer patterns in China. ASR, Age-standardized rate; BMI, body mass index; HBV, hepatitis B virus; HCV, hepatitis C virus; HPV, human papilloma virus; *H pylori*, *Helicobacter pylori*.

Incidence rates of cervical cancer in women and stomach cancers in both sexes are higher than in many high-income countries. Overall cancer mortality is also notably higher; the most frequent deaths are from prostate, lung, female breast, cervix, colorectal, stomach, and liver cancers. Incidence rates of colorectal, prostate, and thyroid cancers have increased in Argentina, Brazil, Chile, and Costa Rica from 1997 to 2008.^7^ The high incidence and mortality of gallbladder cancer in the region, particularly in Chile, is worth noting. Non-Hodgkin lymphoma (NHL) is also among the leading male cancers (Fig 3A).^5,7-15^

#### Arab states of the Persian Gulf.

Most common types of cancer are those typically linked to westernized lifestyles (eg, breast, colorectal) but also leukemia, NHL, and thyroid cancer.^5^ Obesity is an increasing problem in this region. Most of the Gulf countries are among the top 10 countries for obesity rates, and physical inactivity is one of the highest in the world; limited access to culturally acceptable physical activity with a resulting increase of sedentary lifestyle along with an unhealthy diet are contributing factors.^16^

#### Non-EU Eastern European countries and former Soviet republics.

Stomach cancer mortality is high for men and women.^5^ Central and Eastern Europe have the highest lung cancer mortality rates in men worldwide; smoking patterns suggest that this figure is increasing and will stay high for several decades in republics of the former Soviet Union in Central Asia (such as Kazakhstan, Turkmenistan, and Uzbekistan).^17^ This region of the world is characterized by marked variations in the incidence rates of different types of cancers. Examples include a high incidence of bladder cancer among men and thyroid among women in Eastern countries, such as Kazakhstan; high esophageal cancer incidence in Central Asian countries^18^; or increasing risk of cervical cancer in Central Asia, where Kyrgyzstan, Kazakhstan, and Armenia already have high cervical cancer incidence rates.^19^ Mortality for head and neck cancer is almost three times higher in Russia than in the United States, probably related to alcohol consumption along with high smoking rates^18^ (Fig 3B).^10,17-23^

China and India account for 38% of the world’s population. Cancer is the leading cause of death in China; mortality from cancers of the liver, stomach, esophagus, or cervix (common cancers occurring in developing countries) has remained high, especially in rural China, whereas mortality from lung, colorectal, and breast cancer (related to westernized lifestyles) has increased in urban areas. The most common causes of cancer-associated disability-adjusted life-years lost in 2010 were lung and liver cancer.^18^ The age-standardized smoking prevalence in 2015 was 38% for males and 2.2% for females (Fig 3C).^5,8,18,24-26^ In India, 50% of all cancer deaths are due to oral and lung cancer in men and cervical and breast cancer in women, with 40% of all cancers attributable to tobacco use, mainly in the form of smokeless tobacco; interestingly, the world’s highest incidence of cancers of the hypopharynx and of tongue in men, as well as high incidence of gallbladder cancers, are found in northern India.^18^

#### Africa.

The most common cancers in 2012 were prostate (16.4%), liver (10.7%), and Kaposi sarcoma (6.7%) in men and breast (27.6%) and cervical cancer (20.4%) in women. Breast cancer has a high mortality in women, given its lower incidence than in other parts of the world, because of the poor survival, mainly due to of late-stage presentation of patients.^27^

### Risk Factor Patterns

Following the methodology developed for the European Code Against Cancer,^4^ adding, dropping, or modifying recommendations and supporting information (questions and answers) could be done according to the regional context of these areas. This depends on the prevalence of risk factors, as described here, the state of development of the health system, and the related ability to implement preventive interventions of different types. Here we raise some considerations that could influence the composition of a Code Against Cancer in different regions of the world. Some of the European Code recommendations may not apply elsewhere, and their inclusion in a Code Against Cancer in other regions would be confusing. Examples might include radon, the use of sunbeds, or the use of hormonal replacement therapy. In contrast, some regions have specific exposures of importance that are far less relevant in Europe. A careful assessment would be required, therefore, of the need to include different messages on regional specifics in areas such as dietary practices; foods and beverages, including alcohol (eg, in the Arab states of the Persian Gulf); and consumption of very hot drinks (including South American maté).^14^ In contrast to the above areas of variation, it is unquestionable that recommendations on tobacco use, maintaining a healthy body weight, and physical activity will be relevant in all regions. In regions where obesity is endemic, for example in the Arab states of the Persian Gulf, the use of taxation and other policy interventions on sugar-sweetened beverages and junk foods merit evaluation. Recommendations on safe sexual behavior and vaccination of women, and possibly men, for preventing high-risk human papilloma virus (HPV) infection or *Helicobacter pylori* (*H pylori*) eradication programs will have a greater effect in regions with a high prevalence of infection. Infection-related recommendations will certainly be important in Latin America (14.4% of all cancers *v* 7% in the EU),^6^ in particular, where HPV (12.3% to 20.4%) and *H pylori* (50% to 95%) infections are highly prevalent.^7^ In China, chronic infections account for 29.4% of all cancers (mainly hepatitis B and C and *H pylori*)^24^; *H pylori* is also highly prevalent in parts of the Central Asian republics and some regions of Russia.^18^ Despite the observed declines in incidence, the absolute burden of stomach cancer remains particularly high in several Asian and Latin American countries.^5^ Similarly, cervical cancer mortality rates have been increasing in some West and Central Asian countries among younger women.^18^ In sub-Saharan Africa, at least 32.7% of cancers are caused by infectious agents (excluding some resulting from HIV).^27^ In addition, the high HIV-AIDS prevalence in Africa increases the risk of a variety of cancers, including Kaposi sarcoma, NHL, Hodgkin lymphoma, and several HPV-related cancers. Burkitt’s lymphoma is a common cancer of children in parts of tropical Africa; in central Africa, the majority of cases are associated with Epstein-Barr virus.^27^ Regarding environmental and occupational exposures, strict regulations such as the ones applied by the EU countries are not always implemented in other parts of the world, where industrial and agricultural pollution may be of concern.^12,17,28^ Susceptible populations deserve special attention, such as those living near polluted areas, the informal sector, and children.

The implementation of secondary prevention is extremely varied across the world. What is certain is that some screening methods considered of value in high-income countries in Europe may not be immediately applicable or feasible in limited-resource settings.^12^ The challenges and solutions will require careful adaptation to the specific situations being faced, including available resources and health services infrastructure. For example, in Latin America, several countries have established or are considering mammography screening, but in general participation rates are much lower than the coverage recommended by the WHO,^29^ and access to screening services is not equitable. Similarly, cervical cancer mortality has not decreased at the same rate as in other areas in many countries of the region that have cervical cytology screening programs as a main strategy for cervical cancer control, probably because of suboptimal coverage and follow-up coupled with disparities in screening compliance and poor access to health care,^7,8^ in addition to inherent limitations of cytology screening (low sensitivity). Breast cancer is the most frequent cancer in females in the Arab states of the Persian Gulf, where the majority of patients tend to be diagnosed at a late stage; yet, cervical cancer is less common than in other regions. There are currently no organized screening programs at the national level in this region.^16^ In Eastern Europe and the former Soviet republics, public awareness of the benefits of mammographic screening is low^18^; cervical cancer screening is mainly opportunistic and has generally low or unreported coverage, inefficient call-recall systems, and lack of quality assurance. In China, cancer screening programs are not yet available to the entire population but only to certain urban populations or subgroups with high rates of opportunistic screening. Colorectal cancer screening programs are absent or infrequent in the regions mentioned above. In Latin America, although most countries have fecal occult blood tests or fecal immunologic tests available in the public and private sectors, colonoscopy examinations are mostly available in the private sector. Only Uruguay and Argentina have implemented national screening programs.^7^ In general, the frequency of uptake of available cancer control interventions may be impaired by traditional beliefs and practices in some cultures.^18^ Similarly, faith in traditional medicine may have a negative effect on the timely presentation for medical examination and adherence to treatment even after a cancer diagnosis. The factors affecting more specifically the participation in cancer screening programs are many and varied, including lack of knowledge, cultural attitudes toward screening tests, and lack of encouragement by family members and physicians. Inexpensive or less-complex cancer screening technologies may be considered for some populations with low access to health care (eg, self-collected sample for HPV DNA testing, visual inspection of cervical or oral cavity malignant lesions, or clinical breast examination as an early detection strategy).^29^

Notwithstanding the specific topics covered in a Code, of utmost importance is the communication and dissemination through the most effective channels regionally. For example, cultural adaptation of the messages to match ethnic diversity is important; there are > 370 million self-identified indigenous peoples in 70 countries around the world (almost 6% of the global population), who form approximately 5,000 distinct groups and speak > 4,000 of the world’s 7,000 languages. The biggest concentration is in Asia and the Pacific (an estimated 70%); in Latin America alone there are > 400 groups, each with a distinct language and culture, composing 10% of the total population.^30^ Furthermore, future regional Codes may contain a multilayered structure of recommendations to allow the targeting of different audiences according to the local needs, for example to health professionals in addition to the general public. Recommendations to clinicians and public health decision makers may provide effective messages for helping individuals modify their risky behaviors, as well as promoting early detection programs, along with advocating for the implementation of evidence-based and culturally, socially, and economically suitable medical interventions that strengthen national health systems. New technologies such as mHealth can help with hard-to-reach populations (eg, indigenous populations, usually geographically isolated and with fewer resources available) or may allow recommendations to be directed to health professionals using telemedicine in remote areas.

In conclusion, in May 2017, the Member States of WHO adopted a resolution on cancer prevention and control on the occasion of the World Health Assembly. The resolution emphasized the multipronged approach needed across prevention, early detection and diagnosis, treatment, and palliative care.^31^ We propose the development of a World Code Against Cancer as a set of evidence-based recommendations constituting a valuable evidence-based tool to reduce the global cancer burden. We propose the ECAC and its related IARC methodology as a starting point to adapt the approach to other regions of the world. Differences in the prevalence of risk factors, patterns of cancer, and the development of the health care infrastructure across different regions, outlined above, underscore the importance of an in-depth appraisal of recommendations on primary and secondary prevention of cancer, to permit a suitable tailoring to the different contexts and adaptation to the local needs and priorities. The development of a set of cancer-prevention recommendations suited to the regional economic, social, and cultural conditions may offer an exceptional public health tool to support governments in the implementation of their cancer control strategies, in part in response to the resolution mentioned above. Support from authoritative regional leaders in cancer prevention and control allows regional ownership of the recommendations and may help to secure the highest acceptance and uptake both by the public and among those working in the health system. Involvement of scientific and civil society networks to ensure the most suitable dissemination and advocacy, for instance via cancer leagues and patient associations, is key in the success of seeing the recommendations implemented. Finally, a call for fundraising to enable this plan to be implemented provides an excellent opportunity for donors and other stakeholders to build much-needed momentum in cancer prevention and control.
